# Evaluate a comprehensive geriatric assessment service framework targeting frail older people who had high risk of requiring long-term care services in Japan: a community-based pilot study

**DOI:** 10.1186/s12877-024-05200-0

**Published:** 2024-08-01

**Authors:** Kanae Sato (Osaki), K. A. T. M. Ehsanul Huq, Kana Kazawa, Madoka Kawai, Michiko Moriyama

**Affiliations:** 1 Graduate School of Biomedical and Health Sciences, Hiroshima University, Kasumi 1-2-3, Minami-Ku, Hiroshima, 734- 8553 Japan; 2 Department of Nursing, Yasuda Women’s University, 6-13-1, Yasu Higashi, Asaminami-Ku, Hiroshima, Hiroshima, 731-0531 Japan; 3 Department of Nursing, Faculty of Health Sciences, Okayama University, 2-5-1 Shikata-Cho, Kita-ku, Okayama, 700- 8558 Japan

**Keywords:** Frail older adults, Kihon checklist, Comprehensive geriatric assessment, Referral service, Care-needy conditions

## Abstract

**Background:**

Frailty has become a key concern in an aging population. A comprehensive geriatric assessment (CGA) service framework was developed and evaluated aiming to target and connect frail older adults who are at high risk of requiring long-term care services.

**Methods:**

A community-based pilot study was conducted in fiscal year 2016 and 2017 in Kure city, Hiroshima, Japan. Participants aged 65 and over living in Kure city, and 393 persons were extracted from the Kihon Check List (KCL) responses. Among the eligible individuals, 101 consented to participate and received CGA and referred to services based on individual health needs. The efficacy was evaluated by referral rate of services, continuity of the service usage, evaluation of participant’s health condition and the quality of life (QoL) after the 6-month follow-up.

**Results:**

Ninety-nine (98.0%) participants needed support for the instrumental activity of daily living, 97 (96.0%) were categorized as locomotive syndrome, and 64 (63.4%) had a depressive tendency. Afterward, 60 participants (59.4%) subsequently accepted the referral services, however, 34 (33.7%) used the services and the remaining 26 (25.7%) did not use the services. The health condition improvements in the service-uses group were statistically significant (*p* < 0.001), however, QoL score did not change between the baseline and 6th -month.

**Conclusion:**

KCL extracted high-risks older people, and CGA revealed related diseases and health conditions. However, the high refusal rate of referral services indicates a necessity to modify the service framework such as by collaborating with community general support centers, which could increase the efficacy of service framework.

## Background

Worldwide, population aging is accelerating rapidly. According to World Population Ageing in 2017, there will be estimated over 2.1 billion older people globally by 2050 with a profound impact on health and social care planning and delivery system. The most problematic expression of population aging is the clinical condition of frailty [1]. Frail older adults need to be provided health services to prevent premature death [2] and negative health outcomes such as falls [3], fractures [4], disability [5] and dementia [6].

The aging population of Japan is increasing rapidly than any other country in the world. In Japan, 27.3% of the national population were 65 years and over in 2016 and Kure City had a population of 33.7% at the same age group, which was one of the most aging cities in Japan [7, 8]. Therefore, frailty has been gained attention as a top priority in Japan. The prevalence of frailty in Japan is increasing with age and reported 7.4% aged 65 years and older, 20.4% aged 80–84 years, and 35.1% aged 85 years and older [9]. The universal long-term care insurance (LTCI) system launched in 2000 to support comprehensive care for older people, 18.3% of Japanese people aged 65 years and over used these services in 2018 [10–12]. LTCI system covers all people aged 65 years or over and people of 40–64 years who develop aging-related diseases such as rheumatoid arthritis or terminal cancer. Under the LTCI system, long-term care services are provided for care-needy condition for their daily life activities. This supporting system enables users to choose their services and service providers and receive integrated medical and welfare services. They provide home-visit care, rehabilitation day services, day care, residential and in-facility services. These services are provided by various organizations such as private companies, and co-payment is set as 10% (20% for income above a certain level) [13]. Therefore, the cost of LTCI has increased rapidly, which jeopardizes the system’s survival, and financial resources are being compromised [11].

Frailty, however, is considered to have many complex components, making it essential to concentrate on managing and supporting individuals [14]. The elements of frailty compose physical, mental, psychological and social aspects. Physical discomforts such as hearing loss, difficulty in walking, arthritis and cardiovascular diseases appear with ascending age. Psychological problems such as cognitive impairment and depression developed due to confrontation with different situations of loss and shifting their home for long-term care facilities. Cognitive impairment sometimes develops dementia and also related to physical, social and psychological frailty. Social problems such as living alone and economic deprivation leading to frailty. These are interconnected and affected by illness and contribute to disability [15]. Therefore, the Ministry of Health, Labour, and Welfare of Japan started a national health initiative Kihon Checklist (KCL) in 2006 to identify persons aged 65 years and over who are at risk for getting care-needy condition and require LTCI [16]. As KCL is a self-reporting survey, it is necessary to identify the causes and factors of frailty and find a solution for them. The comprehensive geriatric assessment (CGA) tool has been developed with the goal of assessing the condition of frail older adults and providing services based on the results in delivering the older people medical care [17, 18].

Therefore, the researchers with the municipal government developed a project targeting frail older adults at high risk of getting use of LTCI services, further assessing their condition, and referred them to formal and/or informal services according to their needs to prevent them moving to the care-needy condition under LTCI. The purpose of this study was to evaluate/ develop a CGA service framework including introducing a screening, assessment, and referral service targeting frail older adults who were at high risk of requiring LTCI services.

## Methods

### Study design and participants

A community-based pilot study with a pre-and post-design was carried out in fiscal year (FY) 2016 and 2017 in Kure-city, Hiroshima, Japan. Since this study was implemented as a municipal government pilot project, sample size was set as 50 per FY based on their budget. The municipal government administered KCL by postal mail to all the citizens aged 65 years and over to identify the frail or at-risk individuals in 2014. Participants were screened and included those who were identified as frail or at-risk individuals and living in Kure-city at the commencement of the project. The inclusion criteria were the frail older adults aged 65 years and over with declined locomotor function, being homebound and had depressive mood or declined in cognitive function, and willing to participate. These three combination of KCL domains were found to be more reported to introduction of LTC certification in the residents of Kure city [19]. Participants who had certified as requiring the LTCI services, hospitalized, and enrolled in government prepared frailty prevention programs were excluded. After obtaining informed consent at participants’ home, they were assessed using the CGA algorithm.

### The project team and quality assurance

The team was comprised of four registered nurses and one nursing assistant, and they were hired for this study. The nurses received an orientation of the project, education about CGA, frailty and disease management and community services from the researchers prior to initiation of this project for highest quality assurance. In addition, the nurses and the researchers who were specialized in chronic care and gerontological nursing conducted workshops before starting the project and continued monthly case conferences and reviewed the intervention during the project.

### Study procedures

1. CGA to identify the health care needs.

The CGA algorithm assessed first cognitive function and dementia, followed by depression, nutritional status, homebound, locomotive function, normal and instrumental activities of daily living (IADL), and disease conditions. The settings for each scale were adopted as follows:

#### CGA and its criteria

##### Cognitive function

Mini Mental State Examination (MMSE) [20].

Out of a maximum of 30 points, it was judged that the higher the score, the higher the cognitive function. Generally, 0 to 17 points were judged as severe cognitive decline, 18 to 23 points were judged to be mild cognitive decline, and 24 to 30 points were judged to be no cognitive decline.

2) **Problem behavior:** Dementia Behavior Disturbance Scale (DBD scale) [21].

Hearing from family members living together when MMSE score was 23 or less. If there was 1 or more points, it was judged that there was a problematic behavior.

3) **Geriatric Depression Scale:** Depression Scale for the Elderly Shortened Version-Japanese Version (GDS-5) [22].

Depressive tendency was judged with 2 points or more.

4) **Vitality Index:** motivation-related to activities of daily living (VI) [23].

Hearing from family members living together when 2 points or more on the depression scale. Out of 10 points, 7 points or less was considered as low motivation and it was judged that attention was required for life prognosis.

5) **Nutritional status:** Mini Nutritional Assessment (MNA) [24] Simple nutritional status assessment sheet.

Screening was a perfect score of 14 points. If the score was 11 or less, 12 items were added and assessed, and a total of 17 to 23.5 points were judged as “risk of malnutrition”, and less than 17 points were judged as “malnutrition”.

6) **Locomotive Syndrome Degree:** Locomotive Degree Test [25].

In consideration of the safety of the subject, a 2-step test, 25 items and 5 stages of questions were conducted in this order.

- Two-step test: A value obtained by carefully dividing the stride length of two steps were judged to be “locomotive degree 1” if it was less than 1.3, and “locomotive degree 2” if it was less than 1.1.
- Stand-up test: If one foot couldnot stand up from a platform with a height of 40 cm, it was judged as “Locomotive degree 1”, and if both legs couldnot stand up from a stand with a height of 20 cm, it was judged as “Locomotive degree 2”.
- Locomotive questionnaire: A score of 7 or higher was judged as “Locomotive degree 1”, and a score of 16 points or higher was judged as “Locomotive degree 2”. The higher the score, the lower the movement function.

7) **Instrumental Activity of Daily Living (IADL) :** means, intellectual, and social activity [26].

The lower the score out of 0 to 13 points, the less independent living behavior. The activities included managing finances, taking medications, housekeeping, food preparation and laundry.

8) **Activities of Daily Living (ADL):** Barthel Index [27].

A perfect score of 100 indicated activity ability; however, a perfect score did not mean that the participant could live alone.

When the assessment score of each scale fell below/over the cut-off value, the nurses conducted additional assessments, such as nutritional status by assessing oral function, swallowing, underlying diseases, meal per day, total energy intake, nutritional balance, and risk of abuse not taking care properly by the family. Additionally, the nurses recorded information about the history of hospitalizations with medication used, falls and bone fractures, financial and family situation. The nurses also monitored and evaluated the participant’s vital signs, symptoms, and home environment for any possible risks for frailty.

##### Referral services to the participants

Based on CGA, nurses referred the participants to healthcare services, if they needed. The assessed conditions and referred serviced by the nurses are listed in Table 1. This procedure took about 60 to 90 min. The nurses provided decision-making support to the participants and their families, so that they could decide their priorities and ways to solve their problems. They also provided telephone follow-ups for 1 to 4 times per month based on the participant’s physical and mental conditions up to 3 months. If participants were not continuously connected with their healthcare services, nurses coordinated with the services and supported dealing with the issues. Nurses checked the participants’ ‘Behavioral and Psychological Symptoms in Dementia (BPSD)’ symptoms or problematic behaviors. If those behaviors were absent, they used an illustrated pamphlet to explain the condition of the participants according to their level of understanding and obtained their consent. In case of cognitive impairment, nurses involved family members and introduced them to the community general support center for LTCI services. As adult literacy rate in Japan nearly 99% in 2021 [28] and memory domain of MMSE is less affected by education [29], we did not modify the MMSE scale by education level for our study.

If the participants were physically frail, most of the services providers arranged transportation for using services or service providers provide home-care services at patient’s home. For that, to prepare for the use of the service at patient’s home, a system is in place in which a person in charge visits the home of the elderly and provides services at home. Study nurses contact community general support center (CGSC) who needs referral services. Staff of CGSC and care manager assess the patients immediately and care manager make a care plan and contact with referral service providers and service started immediately. The coverage of the service depends on the certified care-needy level. If the person’s condition is assessed as no care-needy level and not certified, the service is not covered by the LTCI, and the patient needs to pay. Even if he/she is certified, there is a co-payment from 10% up to 20% depending on their economic condition. This co-payment sometimes makes unwillingness to use the service.

**Table 1:** Participants’ condition and the referred services they received

In case of cognitive function decline,
- referred to local elderly community general support center for preventive and care management.
In case of depressive condition,
- the nurse provided continuous telephone counseling and referred to a regional mental health center.
In case of nutritional state declined,
- introduced to dentists, primary physicians, specialists, home-help services, food-delivery services, or community activities according to their needs. The nurse also provided educational guidance for the right meals.
In case of locomotor function declined,
- referred to community rehabilitation services to receive health guidance for exercise or medical intervention for orthopedic support.
In case of IADL/ADL declined,
- introduced to the local community general support centers for home help services.
In case of homebound conditions,
- referred to the specialists (incontinence, rehabilitation, etc.) or community services such as a day service and community activities.
In case of economic difficulties, housing issues, or domestic abuse,
- referred to the local government and/or social welfare council.
In case of diseases were not managed well,
- the nurses provided disease management education. Concurrently, the nurses shared the assessment results with participants primary physicians.

### Evaluation of the progress and study outcomes

#### Nursing record for objective evaluation (during 6 months period)

After 6 months, the nurses visited the participant’s houses and collected data on the service usage status and evaluated the health condition of the participants. Nurses described participants’ any changes during 6 months on nursing records. Based on the records, nurses evaluated the physical, mental and social status and categorized the status into three groups (deterioration, no change, and improvement) of the participants: If the participants’ physical aspects (i.e. improvement of edema, weight optimization with proper diet and water, positive lifestyle behavior changes), mental (i.e. increase of positive thoughts, diminish of negative emotion) and social aspects (i.e. increase family and friend’s relationship, increase the frequency of going out) were assessed as “improved”. If their physical condition got worse like onset of new diseases/complications (i.e. pneumonia, heart failure, diabetes/elevated blood glucose), fall and fracture, hospitalization, increase of depressive tendency, and decrease in social interaction with others, we considered their condition as “deteriorated”. When their physical, mental, and social aspects remain the same or no improvement, we considered it “no change”. The evaluation was conducted with a team of researchers. This classification was discussed by the nurses and the researchers based on the nurses’ progress records and agreed.

### Outcome variables

The study outcomes were evaluated by (1) referral rate to services, (2) continuity of the service usage, (3) participant’s health condition, and (4) the quality of life (QoL) after 6-month follow-up. Moreover, the feasibility of this project was qualitatively evaluated by interviewing the service providers involved in this project.

### Questionnaires for evaluation

The standardized EuroQol-5 dimensions-5-level (EQ-5D-5 L) questionnaire [30] was used to assess the QoL, which was developed for measuring 5 dimensions including mobility, self-care, usual activities, any discomfort, and anxiety/depression. Each dimension had 5 levels: no problems, slight problems, moderate problems, severe problems, and unable to/extreme problems. Values close to 1.0 are considered high QoL.

### Qualitative evaluation

A semi-structured interview was administered by the researcher (1st author, a doctoral nursing student) to nurses (*n* = 4), care managers from 8 community general support centers (*n* = 29), and primary care and hospital physicians (*n* = 7) who were involved in this project.

The interview guide was (1) an evaluation of the identification method of the participants, (2) an evaluation of methods of service matching and referral, and (3) an evaluation of this project.

### Data analysis

For descriptive statistics, mean and standard deviation of each scale of CGA was calculated. A chi-square test was performed to see the changes in participants’ health condition and service use. t-test was used to assess the score changes in QoL between those who used the referred services and those who did not. The Statistical Package for the Social Sciences (SPSS) version 25.0 (IBM, USA) was used for analysis, and *p* < 0.05 was set as significant.

Qualitative interviews were recorded and transcribed, and contents were extracted related to the evaluation. To ensure accuracy of the contents, results were shown to interviewees during the project reflection meeting for confirmation.

### Ethical consideration

This project was conducted in accordance with the personal information protection ordinance of Kure City. The researchers developed the service framework and supervised the project implementation, and received and analyzed the anonymous data upon completion of the project. This study was approved by the Ethics Committee of Hiroshima University, Japan for the secondary use of the data and qualitative interview (Approval No. E-524) and registered with the UMIN Registration (UMIN000032123, dated: 05/04/2018). The nurses obtained written informed consent from all the participants before enrollment.

## Results

### Recruitment and registration

**Fig. 1:**
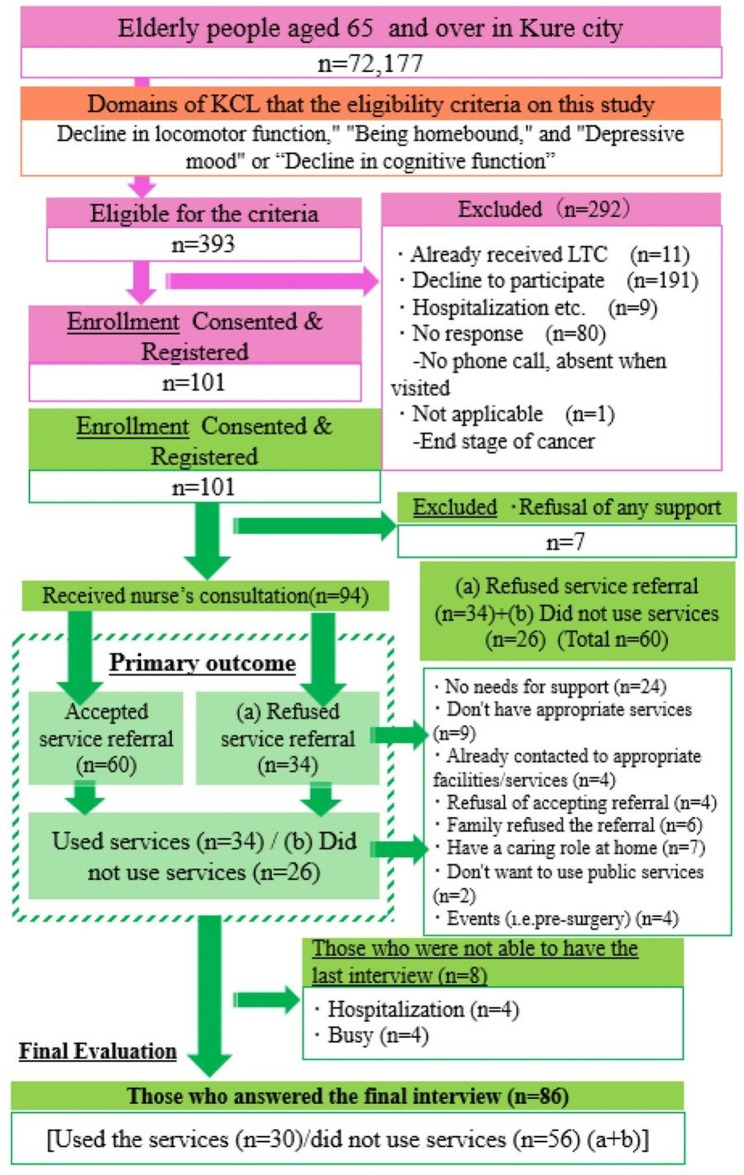
Flow chart of study activities. n = number of the participants; LTC = Long-term care; CGA = Comprehensive geriatric assessment

A total of 393 participants were eligible, and 101 enrolled (50 out of 276 in FY2016 and 51 out of 117 in FY2017). Seven participants refused to receive any support, 94 continued consultations for referral to healthcare services. As 8 participants failed to response, 86 participants were finally evaluated for pre- and post-analysis. The service utilization rate for those who asked for support was 34.9% (Fig. 1).

### Baseline characteristics of the participants

The average age of 101 participants was 80.6 ± 6.0 years (min-max ages were 67–95 years) and 63 females (62.4%). Participants of 98.0% needed support for IADL, 96.0% had locomotive syndrome, 63.4% were categorized as a depressive tendency by GDS-5, 58.4% needed support for ADL, 32.7% were categorized as a risk for undernutrition by MNA, and 25.7% categorized as cognitive impairment by MMSE (Table 2).

**Table 2:** Participants with positive results on each scale of CGA tools (*n* = 101)

Scales	*n* = 101 (%)	Positive (%)	Mean ± SD	Min/Max	Normal range
Mini-mental State Examination (MMSE)		26 (25.7)	25.1 ± 4.2	7/30	Above 24
Dementia Behavior Disturbance Scale (DBD)	*n* = 15	11 (10.9)	5.6 ± 5.8	0/18	0
Geriatric Depression Scale (GDS-5)		64 (63.4)	2.3 ± 1.5	0/5	Under 1
Vitality Index (VI)	*n* = 33	7 (6.9)	8.8 ± 1.3	6/10	Above 8
Mini Nutritional Assessment (MNA)		33 (32.7)	11.9 ± 1.8	6/14	12–14
Homebound (Did you go out in the past week? )		11 (10.9)			Yes
Instrumental Activities of Daily Living (IADL)		99 (98.0)	7.0 ± 3.4	0/13	13
*instrumental self-maintenance*		74 (73.3)	3.0 ± 1.8	0/5	5
*the intellectual activity scale*		69 (68.3)	2.5 ± 1.3	0/4	4
*the social role scale*		92 (91.1)	1.5 ± 1.3	0/4	4
Activities of Daily Living (ADL)		59 (58.4)	92.5 ± 11.3	40/100	100
Locomotive syndrome		97 (96.0)			
*Locomotive syndrome 1*		3(3.0)			
*Locomotive syndrome 2*		94 (93.1)			

n = number of the participants; SD = standard deviation

Sixty out of 101 participants (59.4%) agreed to be referred to the services (Fig. 1). The most commonly referred participants to the community general support center (*n* = 51; 85.0%), followed by medical institutions (*n* = 17, 28.3%), 24 (23.8%) started to use LTCI services.

### Referral to health care services

The most common service received by participants (*n* = 18) was daycare service introduced with deficiency areas of ALD/IADL, locomotive syndrome, and depressive mood. Home renovation such as added handrails was introduced (*n* = 10) for IADL/ADL difficulty and/or locomotive syndrome. However, 26 participants did not use the referred services. Consequently, 34 participants used services (Table 3).

**Table 3:** Referred services based on CGA evaluation results (*n* = 60), n (%)

	Comprehensive Geriatric Assessment (CGA)								Locomotive syndrome
	MMSE	MMSE+DBD	GDS-5	GDS-5 + VI	MNA	Homebound	IADL	ADL	
Connected cooperation destination (*n* = 60)	16 (26.7)	9 (15.0)	39 (65.0)	7 (11.7)	19 (31.7)	10 (16.7)	60 (100)	35 (58.3)	59 (98.3)
Received services (*n* = 34)	7 (43.8)	5 (55.6)	22 (56.4)	4 (57.1)	12 (63.2)	4 (40.0)	34 (56.7)	21 (60.0)	34 (57.6)
Day service/daycare (*n* = 18)	4^†^	3^†^	12^†^	1	6^†^	1	18^†^	12^†^	18^†^
Home renovation (*n* = 10)	1	1	6	1	1	0	10	8	10
Informal service (i.e., talked voluntarily) (*n* = 4)	0	0	3	0	1	0	4	0	4
Medication adjustment at clinics (*n* = 4)	1	0	3	0	1	2^†^	4	3	4
Home visiting nursing or medical services (*n* = 3)	2	2	1	0	2	1	3	2	3
Home help services (*n* = 2)	1	1	2	1	2	0	2	1	2
Dietary supplement prescription at clinics (*n* = 2)	1	1	2	1	2	0	2	2	2
Did not use service (*n* = 26)	9	4	17	3	7	6	26	14	25

n = number of the participants; %=percentage; MMSE = Mini-mental State Examination; DBD = Dementia Behavior Disturbance Scale; GDS = Geriatric Depression Scale; VI = Vitality Index; MNA = Mini Nutritional Assessment; IADL = Instrumental Activities of Daily Living; ADL = Activities of Daily Living; †=The most number service at each CGA assessment item

One person had multiple areas of positive score on the scales and used multiple services

### Comparison of participant’s health condition after service usages

Among all (86) participants, 30 (34.9%) used and 56 (65.1%) did not utilize the referred services.

Of 7 (8.1%) with deterioration, 2 (6.7%) used services, and 5 (8.9%) did not. More than half (59; 68.6%) of the participants’ health condition did not change; 14 (46.7%) in the service users and 45 (80.4%) in the non-service users. However, 20 (23.3%) of the participants improved, 14 (46.7%) from service users and 6 (10.7%) from non-service users, significant higher rate of improvement was obvious (*p* < 0.001) between the service and non-service users (Table 4).

**Table 4:** Evaluated of health condition either used or not used referred services

Variables *n* (%)	Deterioration *n* (%)	No change *n* (%)	Improvement *n* (%)	*p*-value
All participants (*n* = 86)	7 (8.1)	59 (68.6)	20 (23.3)	
Used services: 30 (34.9)/ did not use services: 56 (65.1)	2 (6.7)^§§^ / 5 (8.9)^§^	14 (46.7) / 45 (80.4)	14 (46.7) / 6 (10.7)	< 0.001

n = number of the participants; %=percentage

p = < 0.05 considered significant; §=Significantly lower in the chi-square test; §§=Significantly higher in the chi-square test

We explored the differences in the baseline characteristics between those who used and not-used the referred survices and found that there were significant differences of participant’s body weight and environment among two groups (Table 5).

**Table 5:** Baseline characteristics between used or not used referred services

	Used services *n* = 30	Not use service *n* = 56	*P*-value
Age (years)	82.3 ± 5.1	80.1 ± 6.1	0.101^a^
Weight (kg)	50.7	55.7	0.049*^a^
Body mass index (kg/m^2^)	22.7 ± 4.3	24.0 ± 4.4	0.220^a^
Systolic blood pressure (mmHg)	133.2 ± 22.2	132.0 ± 14.7	0.312^a^
Diastolic blood pressure (mmHg)	66.0 ± 9.2	69.7 ± 11.8	0.386^a^
Heart rate (per minute)	75.0 ± 12.1	72.1 ± 11.0	0.284^a^
Chronic disease; yes/no (n (%))	14 (46.7) / 16 (53.3)	26 (46.4) / 30 (53.6)	1.000^b^
Depression; yes/no (n (%))	19 (63.3) / 11 (36.7)	36 (64.3) / 20 (35.7)	1.000^b^
Homebound; yes/no (n (%))	5 (16.7) /25 (83.3)	6 (10.7) / 50 (89.3)	0.504^b^
Locomotive syndrome; yes/no n (%))	28 (93.3) / 2 (6.7)	52 (92.9) / 4 (7.1)	1.000^b^
History of fall; yes/no (n (%))	12 (40.0) / 18 (60.0)	19 (33.9) / 37 (66.1)	0.641^b^
Poverty; yes/no (n (%))	5 (16.7) ± 25 (83.3)	4 (7.1) ± 52 (92.9)	0.266^b^
Environment^§^; yes/no (n (%))	15 (50.0) / 15 (50.0)	13 (23.2) / 43 (76.8)	0.016*^b^
Hospitalization; yes/no (n (%))	0 (0.0) / 30 (100.0)	1 (1.8) / 55 (98.2)	1.000^b^

§: There were worse situation around their house (e.g. dangerous slope, steep steps and stairs, etc.,)

a: t-test, b: chi-square test, *: significant

There were no statistical significance changes occurred for QoL score after 6 months follow-up (Table 6).

**Table 6:** Evaluation of QoL by whether the service is used at the baseline and in 6th month

Variable	Service use (*n* = 30)	No service use (*n* = 56)	
Number of changes in QoL score	-0.003 ± 0.238	0.041 ± 0.217	0.387^a^

n = number of the participants; a = t-test; Changes = 6 M-Baseline

## Discussion

This study evaluated the efficacy of a service framework as a preventive measure of a municipal government project. KCL extracted frail older people, and CGA thoroughly revealed the health-related causes of frailty. In addition, assessments of participants’ medical, financial, and environmental condition gave a deeper grasp of the context and intervention point.

### Advantage of CGA usage

This study’s CGA results showed multifunctional problems in the participants. CGA provides guidance in planning care for older people. The goals of the CGA include early recognition and improvement of geriatric syndromes and increased survival and QoL for patients [31]. A systematic review revealed that CGA might control older adults care which can improve the care provided to hospital-admitted older people [17]. Another systematic review compared the care based on CGA to usual medical care among the older adults in the community. They observed a lower risk of being admitted in the hospital among the CGA group. However, there were no significant difference for admission in the nursing home or death among groups [32]. According to the study, CGA lowers a patient’s likelihood of being admitted to a nursing home and increases their chances of survival. Therefore, using CGA to identify the health problems of older people with multi-comorbidity is found suitable.

### Efficacy of this project

According to the participants’ evaluation, about a quarter of them were improved, there were significant difference between the service and non-service users. Therefore, there are chances to be spared from deterioration when patients were well-referred to and used services. However, high refusal of referral and use of services indicates that this framework needs to be revised. The majority of older people (80% of the population) in Japan already have access to medical care through the country’s national insurance system [33]; who did not use the services believed that they need no assistance from others. Therefore, they and their family members refused to utilize available services, and some of them had a caring role at their home. Moreover, participants’ physical condition and surrounding household environment might be influenced for not using the referred services.

In our study, we included participants with different health conditions from almost no health issues to severe symptoms. There were a number of participants whose medical conditions were improved, six of them did not even require any health care services. Though they did not use the services, they followed the health education received from the research nurses. Fourteen participants who were improved mainly used services such as home renovation, daycare, talk volunteer, adjustment and packaging of medicine. They were suffering with hypertensin, stroke, diabetes, spinal stenosis and depression. Some participants suffered from chronic conditions such as dementia, depression and underwent stomach surgery due to cancer. Although they received the services, their conditions did not change. Even after receiving the services the participants’ condition deteriorated as one participant was living alone, suffered from heart failure with complication (pneumonia), fell and broke chest bone and was hospitalized. Another participant suffered with depression and developed hyperglycemia due to unhealthy food habits and used day service. Moreover, when nurses met, many of them were already in care-needy condition, and the nurse had to immediately refer them to LTCI services. With regards, it is needed to stratify specific subset of the target population by classifying them based on their disease type and severity to get better outcomes. A study showed how nurse case managers used health data to identify high-risk older people [34]. They provided advanced case and disease management education while observing the outcomes for 2 years. They discovered a substantial decrease in medical and long-term costs, decreased duration of hospital stays and admissions compared to the control group; however, they found no significance in the first 12 months.

### QoL of the participants

The participants who were assessed as support needy also refused to receive services. Individuals who utilized the service were significantly “maintained” in terms of outcome indicators such as less exacerbation of illness and less hospitalization, though the presence or absence of this service use was only marginally connected to changes in health condition and QoL. This study included some factors related to frailty syndromes [35] such as locomotive syndrome [36] and ADL/IADL decline [37] that have been identified as lowering the QoL of the older people.

Although the opinions and requirements of the older adults were taken into consideration and communicated to relevant health care organizations, there was a possibility that the used service content did not suit the older adults, and some participants were unable to resolve their health problems due to economic reasons [38] and environmental factors [39]. As the participants noted, it takes some time to intervene with the older adults to establish a rapport of trust and understand their needs [40], this framework needs to change to a method of approach by a trusted key person in the community.

### Qualitative evaluation of this project

All care managers of the community general support centers stated that it was effective to proactively identify high-risk people before an incident occurred. They found this project had preventive effects on frailty-related events. However, two care managers recommended visiting the older people with the regional care manager, not only by the nurses who were new to the residents, to increase participants’ and families’ acceptance.

Four physicians thanked the project for helping their patients resume treatment. One physician brought up the KCL results’ accuracy, and 3 physicians negatively evaluated the project stating that “*connecting to a service would be economically burdensome for the older people*,* so unless there is a request for support from the person or family*,* it is better to just watch over*”. Even though an economic burden may happen instantly to the elder person to manage their current condition, health professionals should foresee the consequence of delayed outcomes. In addition, they opined that nurses were not familiar with the community, hence gaining trust would be rather difficult for the community people, moreover, 6-month intervention was too short to affect behavior change, which led to a low referral rate of participants. Therefore, further proactive prevention projects need to be conducted to show the effects of this.

## Conclusion

In order to prevent a care-needy condition, we developed a new service framework to proactively identify older people who were at high-risk and referred them to the services as needed. Even though we introduced the framework, we were unable to engage older people in the service system and hence were unable to demonstrate the project efficacy. In light of this, we considered the necessity of this proactive healthcare system. Additionally, the high refusal rate of referral services indicates a necessity for revision in this framework to make it more accessible to the stakeholders. Some healthcare professionals (e.g. physicians) expressed lackluster enthusiasm toward identifying people at high-risk for long-term services. In consideration of therapeutic transcendence, how do the attitudes of healthcare professionals influence the receptiveness of individuals to follow through with referrals and much-needed services, need to explore. This also raises the question of whether motivating healthcare professionals would improve overall willingness to accept referral services. This study provides a well-positioned opportunity to address these questions and examine in greater depth the potential reasons contributing to the low uptake of services. Further intervention research with large sample sizes and randomly allocated participants eligibilities to receive the service and those not eligible to receive will guarantee a high probability of determining the influencing factors for receiving LTCI services and QoL.

### Strengths and limitations

The strength of this study was that nurses assessed the health condition of the participants and that enabled them to receive services. Participants developed awareness and understanding about their frailty status and shared decision-making ability between nurses and the participants and families. Some participants struggled to understand the need for support since they were living their daily life without realizing it, even though they were in a frail state.

This study has certain limitations. Because of the small sample size and of more than half of the participants declined referral services, this could be cause of outcome bias. Around half of the participants’ health condition did not change after getting services; however, more than three quarter of the participants health condition did not change while not using services. We found there were significant improvement between the participants of service user and non-user. The decision to utilize the service or not rested with the participants’ discretion rather than being randomly assigned. Therefore, it remains unclear whether this service influenced the health condition or if it was a result of bias among the participants who opted for it. We could not compare the outcomes between FY 2016 and 2017 due to high refusal rate, short evaluation period, and the study activities were interrupted due to an unanticipated circumstance of a natural disaster.

## Data Availability

All data in this study received permission from Kure City as the insurer’s project. The datasets generated and/or analysed during the current study are not publicly available due to the maintenance of confidentiality of the participants and declarations within the written information that participants had agreed on, but are available from the corresponding author on reasonable request.
